# Nurse Leadership Development: A Qualitative Study of the Dutch Excellent Care Program

**DOI:** 10.1155/2023/2368500

**Published:** 2023-02-25

**Authors:** Eline de Kok, Kirsten Janssen-Beentjes, Pieterbas Lalleman, Lisette Schoonhoven, Anne Marie Weggelaar

**Affiliations:** ^1^Dutch Nurses' Association, Orteliuslaan 1000, Utrecht 3528 BD, Netherlands; ^2^Julius Center for Health Sciences and Primary Care, University Medical Center Utrecht, Utrecht University, Universiteitsweg 100, Utrecht 3584 CG, Netherlands; ^3^Fontys University of Applied Sciences, Ds. Th. Fliederstraat 2, Eindhoven 5631 BN, Netherlands; ^4^School of Health Sciences, Faculty of Environmental and Life Sciences, University of Southampton, Building 85, Southampton SO17 1BJ, UK; ^5^Erasmus School of Health Policy and Management, Erasmus University, Burgemeester Oudlaan 50, Rotterdam 3062 PA, Netherlands; ^6^Tranzo, Tilburg School of Social and Behavioral Sciences, Tilburg University, Professor Cobbenhagenlaan 125, Tilburg 5037 DB, Netherlands

## Abstract

**Aims:**

To understand how nurses perceived the contributions of the Dutch Excellent Care Program, the development of nurses' leadership, and their ability to positively influence their work environment.

**Background:**

Research shows that the nursing work environment influences job satisfaction, retention, and quality of care. Many countries have created programs such as the Excellent Care Program to improve nurses' leadership and facilitate a positive work environment.

**Methods:**

A descriptive qualitative study based on 17 semistructured group interviews (participants *N* = 52) and directed content analysis using thematic coding.

**Results:**

Four program processes contribute to leadership development: (1) nurses taking responsibility for their knowledge and skills development; (2) strengthening organizational structures to improve nursing governance; (3) challenging the status quo with quality-enhancing projects; and (4) enhancing awareness of the supportive role of the nurse manager.

**Conclusions:**

The program supported nurses' leadership development for a positive work environment. The interrelatedness of the four processes enhanced the nurses' ability to solve day-to-day problems and challenge the status quo that influenced working practices. *Implications for Nursing Management*. The findings support making improvements to healthcare organizational strategies to encourage nurses to show leadership in their work environment.

## 1. Introduction

Many countries face difficulties in attracting and retaining nurses [1–4]. Previous studies have indicated the vital role of the work environment in retaining staff [5–8]. The literature shows that improving the work environment results not only in higher job satisfaction and nurse retention but also in better quality of care and patient outcomes [5, 9, 10]. In contrast, staff shortages negatively influence care quality [8, 11] and create a less healthy workforce suffering from psychological (e.g., emotional exhaustion) and physical problems (e.g., heart disease and diabetes) [10, 12].

Thus, it is imperative for healthcare organizations to have a positive work environment (e.g. healthy, supportive, and stimulating). A positive work environment is defined as the inner setting of the organization in which employees work and is the result of respect and trust between employees at all levels, getting recognition for good work, getting support from management, effective collaboration and communication, a safe climate, and a healthy workplace [13, 14]. Although several studies have measured the nurses' work environment [15], research is sparse on how nurses not in designated leadership positions can influence their environment.

Nurse leadership appears crucial to creating a positive work environment [5–8, 14]. According to Wei et al. [8], leadership and the work environment are interdependent. Cummings et al. [16] state that leadership helps nurses ameliorate their work environment. Several authors mention healthcare organizations putting emphasis on improving nurse leadership [17, 18] and investing in leadership development [19] to improve the work environment and nursing practices. Many countries have enhancement programs such as “Magnet Recognition” [20] which is used in many countries (e.g., Australia, Canada, China, Saudi Arabia, Belgium, and the United States) and “Healthy Workplace, Healthy You” [21]. Most programs focus on designated leadership positions, that is, nurse executives and managers [8, 22–25]. Often, they focus on transformational leadership in management [16, 17, 26]. However, nurses not in designated leadership positions (i.e., bedside nurses) also exhibit leadership in practice. This is often described as “clinical nurse leadership” [27–30]. Clinical nurse leaders are able to *“display their beliefs and values related to the quality of care and they interact with patients in a “hands-on” fashion, living out their values in the delivery of clinical interventions”* [29]. In their review, Mianda and Voce [28] illustrate the qualities of clinical nurse leaders and their impact on standards of care. The qualities include their ability to promote change, communicate effectively, and gain support to influence others, as well as their role modeling, approachability and availability to support, advise, and guide. In addition, as Uhl-Bien et al. [31] point out, *“leadership is a collective process flowing through networked interactions”, instead of “only a management function occurring in formal leadership roles and hierarchical structures*”. Therefore, supporting, developing, and stimulating leadership of nurses not in designated positions deserves attention both in practice and in science [31].

The Excellent Care Program (ECP) focuses on nurses working directly in patient care, not in management positions [32]. Developed by the Dutch Nurses Association (V&VN), the ECP aims to help Dutch healthcare organizations create a positive work environment by developing nurses' leadership (Box 1). Between 2009 and 2020, 28 healthcare organizations participated in the ECP, beginning with baseline measurements of (1) nurses' perception of their work environment, (2) organizational structures, and (3) nurse-sensitive patient outcomes [33].

Following the baseline measurements, the organizations received the results of the measurements and recommendations to create a plan for improving the nurses' ability to develop their work environment (see e.g., in [34]). During this process, the Dutch Nurses Association supported the healthcare organization and facilitated an ECP-learning community.

Because ECP has never been studied in terms of nurse leadership, it is not clear if it contributes in any way to the development of nurse leadership. This study aims to fill the gap in the literature by describing how the ECP, according to nurses, contributes to the development of leadership and the ability to influence their work environment.

## 2. Methods

Applying a descriptive qualitative study design [35], we collected data in semistructured group interviews. Group interviews are suitable for exploring a research area as they elicit similar types of information from each participant [36] and give all the participants the opportunity to respond to each other's statements and thus establish a shared opinion. Hence, engagement in the discussions is crucial [37]. We used the COREQ (consolidated criteria for reporting qualitative research) checklist to report methods and findings in this study [38].

### 2.1. Setting

All organizations involved in the ECP were invited to participate (*N* = 28). After four weeks, nonresponding organizations received a reminder by email and were contacted by phone. Organizations willing to participate (17/28) were included in the study (Table 1). Reasons for nonparticipation were as follows: all members steering the local ECP were no longer employed (5); there was no time (2); and an organizational merger or financial hardship caused a stop to the ECP (4).

### 2.2. Sample

Interview participants were selected through purposeful sampling based on a predefined set of inclusion and exclusion criteria. Participants were included if they (1) had an overview of the local ECP and implementation progress; (2) were familiar with nurses' views and experiences of the local ECP; or (3) had close experience with the program. Participants were excluded if they were not involved in the ECP. The organizations invited participants in person or by email, as they knew best who was involved. We asked them to select a heterogenous group of (1) nurses responsible for the local ECP (ECP managers, assistants, expert panel members, and/or members of the nursing advisory board); (2) nurses with different levels of education (vocational degree, bachelor's degree, and master's degree) working at the bedside [39]; (3) nurse managers or directors involved in the ECP (unit managers, staff managers, or nurse directors); and (4) nurses working in other functions (researchers, HR advisers, trainers, or policy advisers) (Table 2).

### 2.3. Data Collection

Referring to the literature, we developed with the whole research team an interview guide (Appendix A) that included organizational factors [6], the “Essentials of Magnetism” [40], and various leadership styles [16]. Two researchers (EdK and KJ or PB) held the first three interviews, which allowed us to evaluate the interview process, the role of the interviewer, and the interview guide. The results provided guidance for further data collection and analysis. One researcher (EdK) held the other 14 interviews. All 17 group interviews consisting of 2–7 participants lasted 60–90 minutes and were held at each healthcare organization. The interviews were audio-recorded and transcribed verbatim. One participant from each organization did a member check of the transcripts. Field notes describing the setting and the observations and thoughts of the researcher were also added to the transcripts to reflect and prevent biases and support memory recollection. Data saturation was reached after 15 interviews.

### 2.4. Data Analysis

We used the thematic coding steps of Braun and Clark [41] to conceptualize collected data, exposing every single sentence, and observation. We began familiarizing ourselves with the data by reading and rereading the transcripts (EdK and KJ). Two researchers (EdK and KJ) independently generated initial codes from the transcripts and discussed these up to consensus with and between researchers (EdK, KJ, and AW). We aimed to formulate codes in the same context as the transcripts to stay closely linked to the data (an inductive approach). Next, two researchers (EdK and KJ) refined the initial coding list by adding new codes or reconstructing existing codes. After this, three researchers (EdK, KJ, and AW) discussed and reconciled coding differences. Next, the codes were merged into clusters and defined in themes and subthemes, still aiming to stay strongly linked to the data. Finally, the researchers (EdK, KJ, and AW) interrogated the themes in-depth and reflected critically on their interrelationships [42]. To ensure rigor and enrich data interpretation, we analyzed the field notes in the same way. Data analysis was conducted in Dutch, using Atlas.ti version 8.2.0 software (ATLAS.ti [43]).

### 2.5. Ethical Considerations

Prior to the semistructured group interviews, participants were informed by letter about the research aim, the voluntary nature of the study, their right to withdraw at any moment, and the confidentiality of the collected data. Before the semistructured group interviews began, participants signed an informed consent form. The Medical Research Ethics Committee of the University Medical Center Utrecht (number 19–183) approved the study. Data were stored according to the Dutch General Data Protection Regulation.

## 3. Results

The ECP baseline measurements gave organizations and nurses insight into perceived characteristics of a positive environment, such as clinically competent peers, clinical autonomy, and control over nursing practice and nursing strategy. This information fed discussions between nurses and management about the baseline outcomes and subsequent recommendations that emphasized the need for ongoing development of a positive work environment. The ECP provided a framework for this. Each organization used its outcomes and recommendations to make an individual plan to enhance nurses' leadership skills to enable them to improve their work environment. Nurses worked on this plan with colleagues, including managers of policy departments (e.g., quality and safety and human resources). Despite individual differences in the plans, four common processes were perceived that, according to the participants, contributed to the development of nurse leadership. We describe these four processes in the following sections:

### 3.1. Taking on Responsibility for Continuous Knowledge and Skill Development

Continuous knowledge and skill development was seen as the most important factor contributing to the leadership that would allow nurses to initiate change, make decisions, and deploy strategies to improve their work environment. Nurses realized that if they wanted to have control over their work environment, they needed the necessary knowledge and skills. They also recognized that if they wanted to focus on gaining knowledge and skills, they would have to take the ongoing development in their own hands. Basing their conclusions on the outcomes of the baseline measurements, management often acknowledged that nursing education should receive more attention in their organizations. They also recognized they could support nurses in organizing this.

Most ECP organizations (11/17) began investing in continuous knowledge and skill development, establishing education and training programs. Team-level education mainly gave nurses information about occupational-specific topics related to nursing practices (e.g., nutritional deficiency risks or palliative care). Organizational-level programs, often conducted by in-house training units or professional training institutes, were deployed to refresh and update nurses on clinical reasoning, guideline development, and communication skills. Clinical leadership was observed in both nursing teams and multidisciplinary group meetings.

Our analysis showed that improving evidence-based practice knowledge and clinical reasoning skills was regarded as the most essential component in developing nurse leadership. According to participants, better knowledge and skills enabled nurses to conduct better conversations on the balance between adhering to standardized procedures versus deviating from them to benefit care quality, for example. Gaining this competence taught nurses how to change practices by challenging the status quo to benefit the patient:

*“We'd agreed to enter the alarm score three times a day for all patients. Last week I heard from a doctor that a nurse had wondered if this was necessary for a certain patient category. The doctor thought so because it was the set rule. Pointing to the literature, the nurse backed up her claim that it wasn't needed for this patient category. She discussed it with her nursing colleagues and ultimately the protocol was adjusted.”*

(RN, researcher and ECP manager, hospital).

### 3.2. Strengthen Organizational Structures to Improve Nursing Governance

ECP baseline measurements enabled organizations to improve nursing governance. Also, the nurses' increased knowledge and skills helped them see their impact on relevant topics, such as e-health solutions, infection prevention, and on-the-job learning. In most organizations, participants felt that nurse involvement in nursing topics deserved more attention. One member of the ECP expert panel noted, “*Nurses were* o*ften talked about instead of with*.” These insights made nurses realize they had to show leadership and strengthen their governance to have a greater influence on their work environment. According to the nurses, improving their position increased their professional autonomy and influence:

*“If we want to say something about [patients' length of stay], we'll make sure that we have our say. We will not necessarily discuss it with the board of directors […]. We'll do it informally through our network or we'll find another way to ensure that we get our message across.”*

(RN, nursing policy adviser, hospital).

Existing structures were reinforced and/or new ones were established in nursing governance, such as nursing advisory boards, platforms, and committees. The ECP framework helped nurses address relevant topics in the governance structures and monitor whether their organizations invested in these topics (e.g., time and money for training programs or nursing research). If the nurses felt that corporate investment could be improved, they spoke up or started projects themselves to realize these topics. Nurses felt it was their responsibility to act and had the professional discretionary space to do so.

As the active participation of nurses increased in the governance structures that influenced their work environment, their visibility also increased in organizations:

*“I've noticed that we're being taken more seriously. […] Far more nurses are interested in our work on the nursing [advisory] board because we're getting more and more people wanting to act as key figures. I think this is because of all of those projects on the wards. Nurses want to know more about what's going on at a higher level. So, you notice that people are really busy with professionalization and thinking about how they can improve their work. I think that's an incredibly good development.”*

(RN, clinical nurse specialist, psychiatric organization).

Working on nursing governance structures was an incentive for nurses to build on their formal and informal networks to rally colleagues' involvement in nursing-related projects. These networks supported the exchange of knowledge, enabling nurses to share best practices and the outcomes of quality-enhancing projects and discuss such issues as retaining nurses, patient-centered quality improvement, nursing research, and education. According to the participants, nurses found these networks inspiring. They learned from each other how to show leadership in daily practice and felt supported in collaborating on nursing-related improvements.

*“We achieved this through the nursing platform. It brings people together. Nurses know each other better now. You used to work only in your own department, and that was it. Now people from other departments work together on [improving the work environment and patient care].”*

(RN, policy adviser and ECP manager, hospital).

### 3.3. Challenging the Status Quo with Quality-Enhancing Projects

The insights into the nursing environment, knowledge and skills, and governance structures stimulated the organizations' effort to develop nurses capable of challenging the status quo. Nurses began leading small improvements on the ward level and became involved in organization-wide projects. Nurses not only identified problems; they also suggested how to solve them. For example, a nurse wanted to improve the patient handover process. Apparently, doctors were careless in completing the handover forms, which meant that nurses often had to ask them to clarify postsurgery treatment. Discussing the issue with the doctors had no effect, so this nurse took a different approach. She made a new form that required doctors to fill in more information. This caused an uproar, and the new form was immediately abolished. But now the doctors filled in the old form properly. With this devious act, the nurse solved a longstanding problem that had caused extra work and distress for nurses unable to provide the best patient care:

*“A bit deviating, yes, but sometimes you have to encourage doctors differently.”*

(RN, unit manager and ECP assistant, hospital).

Successful experiences with quality-enhancing projects gave nurses the confidence, motivation, and validation to continue:

*“Now nurses are more concerned with their further professionalization and think more about how they can improve care. That's nice because I feel like we're constantly reinforcing each other. As an organization, we've ended up in a positive flow.”*

(RN, staff manager and ECP manager, psychiatric institute).

Moreover, organizations became more aware of their vital role in quality-enhancing projects. Now, policy departments (e.g., quality and safety, HR) began collaborating more with nurses, offering more support for their quality-enhancing projects, such as developing formats to help nurses initiate a project or by organizing educational meetings about project management. Hence, they facilitated nurses to take responsibility for managing improvement projects in their work environment instead of merely taking part in projects managed by the policy departments.

### 3.4. Becoming Aware of the Supportive Role of the Nurse Manager

Through their participation in the ECP, all 17 organizations became more aware of the supportive role of the nurse manager in the development of nurse leadership. Participants noted that nurse managers substantially influenced the preconditions of a positive work environment. They could clear budget for quality improvements, create opportunities for continuous knowledge and skills development, and support nursing collaborations throughout the organization.

*“At the very least, the role of the manager is to facilitate so that their nurses can work on improving processes.”*

(RN, nurse and ECP manager, hospital).

For example, managers stimulated nurses to reflect on their work routines, improve work rosters, and enhance capacity management.

According to the participants, the degree to which nurses felt supported depended on the manager's confidence-building behavior. For example, involving nurses actively in decision-making processes and standing up for them in hard conversations with colleagues had a positive effect. Some participants mentioned the importance of having a learning organization culture which allows nurses to make mistakes and learn from them.

*“If you limit nurses and keep them on edge, it gets stressful for them. But if you give them space, you'll see them showing valuable qualities, which otherwise might not come up.”*

(Staff manager, long-term care organization).

Overcontrolling managers who do not give nurses space hindered nurse leadership. However, nurses took alternate paths when they experienced managerial obstacles. The first path sought collaboration with colleagues across departments and/or other nurse managers willing to support their aims. The second path involved experimenting with new routines or interventions that were invisible to their managers. For example, a nurse on the nephrology ward introduced a smaller glass to help patients with their fluid restriction without first discussing the change with her manager. She knew she first had to collect evidence for why implementing the new size glass would make a difference before financial constraints would stop the change (RN, manager and ECP assistant, hospital). Not being open gave nurses the space to experiment and avoided discouragement in their attempts to show leadership.

## 4. Discussion

In this study, we investigated how the ECP contributed to the development of nurses' leadership to improve their work environment. In the process, we assessed whether the stated intention of the ECP to stimulate leadership of nurses not in designated leadership positions was achieved [19]. In our assessment of the plans of the 17 healthcare organizations involved, we noticed that the ECP fosters four processes that influence how nurses, who are not in designated leadership positions, take on leadership in their work environment. The nurses began taking the lead on their knowledge and skill development, the nursing governance structures, and nursing-related quality-enhancing projects. Besides this, the growing awareness of the supportive role of nurse managers helped both organizations and nurses understand the preconditions needed for undesignated nurses to demonstrate leadership.

Based on our qualitative data, we cautiously conclude that the ECP contributes to nurses' leadership development to facilitate a positive work environment. We cannot compare our findings to other programs on nurse leadership, such as Magnet Recognition [20] or “Healthy Workplace, Healthy You” [21], because these programs focus on developing leadership of nurses in designated leadership positions [22–25]. However, we can compare our findings with a quasi-experimental, empirical study in one hospital that also found the ECP “positively affects the nurse work environment, job satisfaction and quality of care” [34]. In recent years, leadership of nurses not in designated leadership positions has gained interest, especially after the review by Cummings et al. [44] on nurse leadership styles, other stands on clinical leadership (e.g., [27, 29, 45, 46], and leadership as practice (e.g., [47–49].

Our study adds to the literature on how nurses develop leadership competence with the ECP to benefit their work environment. Wei et al. [8] also show the relation between nurse leadership and a positive work environment. Previous research confirm the importance of single elements in the development, such as continuous knowledge and skills development [17, 26], the influence of nursing governance structures [50, 51], working on quality-enhancing projects [52], and the role of management [30, 53].

**Figure 2:**
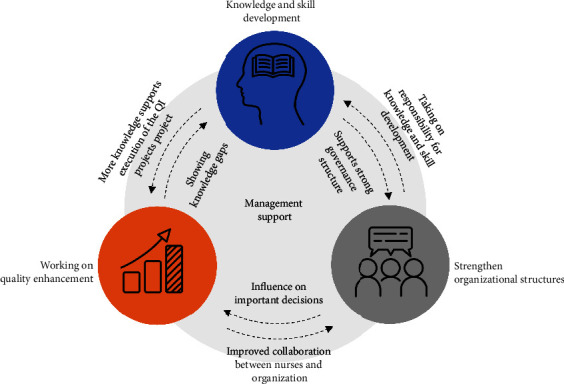
Interrelatedness of processes.

The four processes identified in this study probably strengthen each other.  Figure 2 depicts their interrelatedness as a heuristic model that may need further study.

As nurses gained knowledge and skills, their understanding of the extent to which they had governance over their work environment grew and that helped them develop stronger nursing governance structures. Nurses collaborating on quality-enhancing projects revealed the gaps in their knowledge and skills so that education strategies could be adapted on the organizational level, which in turn gave impetus to their leadership in the work environment. This causality between the findings of our study shows the importance of nurse leadership as it fosters processes directed at a positive work environment [14] that are crucial for job satisfaction and retaining nurses. Keyko et al. [54] state that this leadership provides a higher level of autonomy which correlates positively with work engagement and ultimately improves patient outcomes.

Our study found the supportive role of nurse managers to be a precondition for nursing leadership. However, nurses were not discouraged if they did not get management support. Then, they reflected on their work environment and the line between patient-centered care and organizational regulations. They took the initiative to find practical solutions, challenge set rules, and initiate quality-enhancing projects. They promoted discussion of nurse governance structures and built networks of like-minded nurses. This behavior aligns with the concepts of Gary [55] about “positive deviants” (2013), Meyerson [56] about “tempered radicals,” and Bevan [57] about “healthcare rebels.” All three concepts describe nurses pursuing their nursing ideals to give the best quality of care and the need for deviating behavior when organizational rules/regulations prevent this. The systematic review by Kok et al. [58] finds these interesting concepts for further study. Ethnographic studies would benefit, especially since this behavior is invisible to the rest of the organization [59]. Learning from deviating practices is harder when they are invisible, even if this behavior could have a positive effect on the nurses' work environment and patient outcomes [55, 59].

### 4.1. Strengths and Limitations

This is the first study to provide insights into the contribution of the ECP to nurse leadership development and its constructive effect on the nurses' work environment in Dutch healthcare organizations. The study applied a precisely transparent qualitative method. However, two limitations must be noted. First, as we did not do an effect study, we do not know if the ECP alone contributed to the positive results. Other conditions could have been beneficial, such as changes in financial support or organizational strategies. We tried to overcome this limitation by interviewing ECP participants still working in the organization. However, an effect study on ECP outcomes could shed light on improvements to nurse leadership, retainment, job satisfaction, and care quality.

Second, at the time of the interview, some participating nurses had grown into ancillary functions or designated leadership positions, which can be seen as a result of the ECP. This may have biased the results for nurses who are not in designated leadership positions. These participants could have formed a different view of their organizations than their colleagues working only at the bedside. However, at the beginning of the ECP, most participants worked primarily as bedside nurses. Through the ECP, they took on more responsibility and their leadership might have led to their gaining these ancillary or designated functions.

## 5. Conclusion

According to the experiences of nurses, the ECP contributed to developing the leadership qualities by which nurses influenced their work environment. Nurses took on responsibility for (1) continuous knowledge and skills development, (2) strengthening governance structures, (3) challenging the status quo with quality-enhancing projects, and (4) becoming aware of the supportive role of the nurse manager. The interrelatedness of these processes supported leadership development and its positive effect on the work environment. Nurse leadership development can be stimulated and enhanced diversely by applying several processes at once. This study shows the particular contribution of the ECP to develop nurses who are not in designated leadership positions.

## 6. Implications for Nursing Management

This study shows that a program like the ECP seems useful in helping nurses and organizations create a positive work environment, providing insights into crucial aspects and shedding light on areas of concern. It stimulates nurses who are not working in designated leadership positions to show leadership and enhances collaboration in the organization [34]. Therefore, we recommend investing in developing the leadership of nurses who are not in designated leadership positions [14] to create a positive nursing environment that will also benefit staff attraction and retention [8, 10].

This study reminds nurse managers of their influential position in creating a positive environment [24, 26]. They can ensure that nurses are involved in decision-making, break down the silos in the organization, and develop structures that influence mechanisms that affect patient outcomes [24]. Knowing their strong impact, nurse managers can help nurses develop their knowledge and skills, encourage nurses to cooperate throughout the organization, and engage them in quality-enhancing projects.

## Figures and Tables

**Figure 1:**
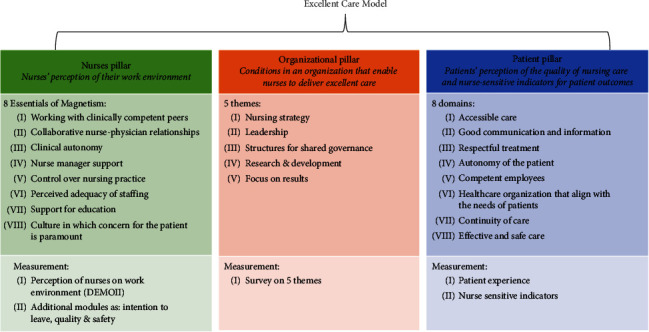
The excellent care model.

**Algorithm 1:**
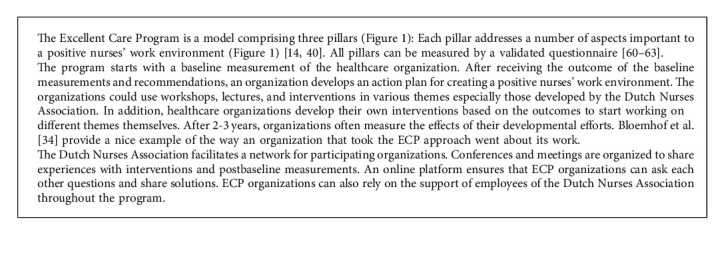
Description of the Excellent Care Program.

**Table 1:** Demographics of healthcare organizations.

	Organization	Environment	Nurses	Start of Excellent Care Program (ECP)
1	Hospital 1	Urban	1349	2016
2	Hospital 2	Urban	1618	2015
3	Hospital 3	Urban	737	2010
4	Hospital 4	Urban	1043	2010
5	Hospital 5	Urban	763	2016
6	Hospital 6	Urban	1034	2010
7	Hospital 7	Urban	290	2011
8	Hospital 8	Urban	625	2010
9	Long-term care organization 1	Rural	2208	2009
10	Long-term care organization 2	Rural	958	2015
11	Long-term care organization 3	Rural	135	2010
12	Long-term care organization 4	Rural	452	2011
13	Long-term care organization 5	Rural	42	2010
14	Psychiatric organization 1	Urban	157	2016
15	Psychiatric organization 2	Rural	210	2013
16	Psychiatric organization 3	Urban	347	2010
17	Rehabilitation center	Urban	98	2016

**Table 2:** Demographics of participants.

Participants (position at start of the ECP)	*N*	Participants (current position)	*N*	Responsibility during the ECP			
				ECP manager/assistant/expert panel	*N*	Member nursing advisory board	*N*
Bedside nurse	23	Bedside nurse	14	ECP manager	15	Member	15
Nurse practitioner	4	Nurse practitioner	4	ECP assistant	4	Vice president	4
Nurse assistant	1	Nurse assistant	1	Member expert panel	5	President	8
Unit manager	8	Unit manager	8				
Staff manager	7	Staff manager	8				
Policy adviser	6	Policy adviser	12				
HR adviser	1	HR adviser	1				
Trainer	1	Trainer	1				
Program manager	1	Nurse director	1				
		Program manager	1				
		Researcher	1				
	52		52		24		27

## Data Availability

The data supporting the findings of this study are available on request from the corresponding author. The data are not publicly available because of privacy and ethical restrictions.

## Appendix

### A. Interview Guide

Start of the interview:
  - *Background questions on demographics including the function and role of the Excellent Care Program.*
Excellent Care Program experience:
  - *What did the Excellent Care Program bring to your institution?*
  - *Why did your organization start the Excellent Care Program?*
  - *What was the work environment of nurses like before the Excellent Care Program started?*
  - *What did you do with your baseline measurement results in the Excellent Care Program?*
  - *What interventions did you implement during the Excellent Care Program?*
  - *What was the work environment of nurses like at the end of the Excellent Care Program, after the interventions were implemented?*
  - *What do you think has helped your organization move toward excellent care?*
  - *After the Excellent Care Program ended, did the quality of care and job satisfaction for nurses improve in any way?*
Nurse leadership experience:
  - *What do you understand by nursing leadership?*
  - *What are nurses doing when they show leadership?*
  - *What do you see happening in the organization when nurses show leadership?*
  - *What is your own role in promoting nurse leadership?*
  - *What has the Excellent Care Program done in terms of nurse leadership?*
  - *Has the Excellent Care Program helped the organization stimulate nurse leadership?*
  - *Which factors do you think have contributed to the development of nurse leadership?*
  - *Are any factors a barrier to the development of nurse leadership?*
  - *What advice would you give to an organization if they want to start stimulating nurse leadership?*
Closing questions:
  - *Have we missed asking any other questions that could help us better understand the outcome of the Excellent Care Program or the development of nurse leadership?*
  - *What was it like for you to take part in this interview, and do you have any questions for us?*
